# Transcriptomic variation of hepatopancreas reveals the energy metabolism and biological processes associated with molting in Chinese mitten crab, *Eriocheir sinensis*

**DOI:** 10.1038/srep14015

**Published:** 2015-09-15

**Authors:** Shu Huang, Jun Wang, Wucheng Yue, Jiao Chen, Sarah Gaughan, Weiqun Lu, Guoqing Lu, Chenghui Wang

**Affiliations:** 1 Key Laboratory of Freshwater Fisheries Germplasm Resources, Ministry of Agriculture, Shanghai Ocean University, Shanghai, 201306, China; 2 Department of Biology, University of Nebraska at Omaha, Omaha, NE 68182, USA

## Abstract

Molting is a critical developmental process for crustaceans, yet the underlying molecular mechanism is unknown. In this study, we used RNA-Seq to investigate transcriptomic profiles of the hepatopancreas and identified differentially expressed genes at four molting stages of Chinese mitten crab (*Eriocheir sinensis*). A total of 97,398 transcripts were assembled, with 31,900 transcripts annotated. Transcriptomic comparison revealed 1,189 genes differentially expressed amongst different molting stages. We observed a pattern associated with energy metabolism and physiological responses during a molting cycle. In specific, differentially expressed genes enriched in postmolt were linked to energy consumption whereas genes enriched in intermolt were related to carbohydrates, lipids metabolic and biosynthetic processes. In premolt, a preparation stage for upcoming molting and energy consumption, highly expressed genes were enriched in response to steroid hormone stimulus and immune system development. The expression profiles of twelve functional genes detected via RNA-Seq were corroborated through real-time RT-PCR assay. Together, our results, including assembled transcriptomes, annotated functional elements and enriched differentially expressed genes amongst different molting stages, provide novel insights into the functions of the hepatopancreas in energy metabolism and biological processes pertaining to molting in crustaceans.

Molting is an essential biological process occurring multiple times throughout the life history of crustaceans. Many behavioral and physiological activities such as growth, development, and reproduction depend on successful molting^1^,^2^,^3^,^4^. Crustaceans experience rhythmic molting cycles, each cycle with four major stages, ecdysis (molting), postmolt, intermolt, and premolt^5^. During ecdysis, individuals must loosen the connectives between living tissues and extracellular cuticles, absorb water to expand the new flexible exoskeletons, and then quickly harden the skeletons for defense and locomotion^6^. During postmolt, the stage immediately following ecdysis, the exoskeleton is hardened via sclerotization and mineralization^6^. Intermolt is the longest period in a molting cycle during which individual crustaceans accumulate energy and gain weight. In premolt, the stage prior to ecdysis, the old exoskeleton is absorbed, and the new exoskeleton will be formed.

Many regulatory hormones/genes and potential pathways that are pertaining to molting have been identified. These include ecdysteriods and crustacean farnesoate (MF), regulatory hormones that promote molting^7^. The primary site for ecdysteroid synthesis is the molting glands (Y-organs or YOs). The neuroendocrine hormones, molt-inhibiting hormone (MIH) and crustacean hyperglycaemic hormone (CHH), which are synthesized and stored in the sinus gland/X-organ complex, were found to inhibit crustacean molting^8^. A number of other hormones/genes, including vitellogenesis-inhibiting hormone (VIH), ecdysone receptor (EcR), and retinoid X receptor (RXR), have been connected to molting. A possible signaling pathway linking MIH to the regulation of ecdysteroidogenesis in decapod crustacean molting gland has been proposed, which include the triggering phase (MIH reduction) and the summation phase (ecdysone synthesis and secretion)^6^. Although much progress has been made in understanding the mechanism of crustacean molting, the studies conducted so far focus mainly on a few important hormones/genes or individual pathways^3^,^6^,^7^,^9^.

The Chinese mitten crab *Eriocheir sinensis*, native to East Asia, is the most important economic crab species throughout the northern and central coastal regions of China^10^. This species has also gained notoriety as an invasive species in Europe and North America^11^. The economic and biological significance of this species makes the Chinese mitten crab a unique system to explore the genes involved in the molting cycle. Periodic molting occurs approximately 18 times during its life cycle^12^. The duration of these molting stages ultimately determines the lifespan of *E. sinensis*^11^,^13^,^14^,^15^. Through each molting cycle, the crab shows saltatory changes or increments in growth and autotomized organ regeneration. Understanding the molecular mechanism pertaining to the molting of *E. sinensis* could promote aquaculture efforts in China and assist its biological control in Europe and North America.

The hepatopancreas plays important roles in carbohydrate and lipid metabolism, nutritional status, energy storage and breakdown in various crustaceans^16^. It is an important organ for the steroid hormone biosynthesis and catabolism related to reproduction system^17^,^18^. The hepatopancreas is also responsible for removing ecdysteroids from the hemolymph and adjusting ecdysteroid changes during the molting cycle^19^,^20^. In addition, large amounts of energy are stored in the hepatopancreas in preparation for molting, reproduction, limb regeneration and other life activities^18^. The hepatopancreas is thus an ideal organ to study transcriptomic changes during molting cycles.

The advent and advance of high-throughput next-generation sequencing (NGS) technology has revolutionized the way we conduct various biological research^21^,^22^,^23^. In this study, RNA-Seq was used to investigate hepatopancreas transcriptome profiles in *E. sinensis*. The specific objectives are (1) assemble and annotate a comprehensive *de novo* transcriptome of *E. sinensis* hepatopancreas, (2) identify differentially expressed genes enriched at different molting stages, and (3) validate expression patterns of important functional genes via quantitative real-time PCR (qRT-PCR).

## Materials and Methods

### Animal Sampling

**Figure 1:**
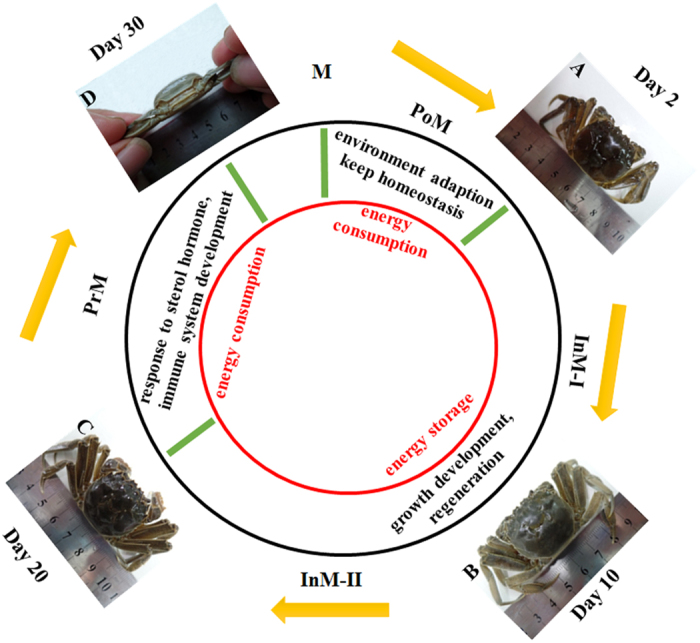
Energy metabolism and physiological responses in a typical molting cycle in Chinese mitten crab. (**A**) Day 2 after molting (postmolt, PoM), soft exoskeleton; (**B**) Day 10 after molting (intermolt-I, InM-I), hardened exoskeleton and cyanish carapace; (**C**) Day 20 after molting (intermolt, InM-II), brownish carapace; (**D**) Day 30 after molting (Premolt, PrM), pleural suture cracks between carapace and abdomen and dark brown carapace. M - molting stage (ecdysis), yellow arrows - molting cycle, green bars - boundaries of different molting stages, black circle - physiological responses, and red circle - energy metabolism pattern in different molting stages.

One-year-old crabs from a full-sib family were sampled from our research facility at the Shanghai Ocean University (China, Shanghai). These individuals were cultured in glass tanks with adequate aeration, temperature (20 °C) and food (twice daily). Based on our previous observations, one molting cycle takes approximately 30 days for a one-year-old *E. sinensis* under glass tank conditions, male crabs grow faster than female, and intermolt (stage) is longer than postmolt or premolt^24^. In our experiment, crabs from four different time points after molting (Fig. 1) were sampled for analysis. Sampling started at the following days: Day 2 after molting (postmolt, PoM) - the exoskeleton of the crabs was soft, and the crabs sheltered in the corner and did not ingest food; Day 10 after molting (intermolt-I, InM-I) - the exoskeleton was consolidated and hardened, the color of the carapace turned cyanish, and the crabs started to ingest food; Day 20 after molting (intermolt-II, InM-II) - the external morphological pattern of the crabs looked similar to that at InM-I stage except that the color of the carapace turned brownish; Day 30 after molting (premolt, PrM) - pleural suture cracks between the carapace and the abdomen of the crabs were present, the shell color turned dark-brown, and the crabs stopped eating. According to Phlippen’s description of molting stages, PoM should correspond to stage B, InM-I and InM-II both correspond to stage C, and PrM corresponds to stage D3 25. Three individual crabs were sampled at each molting stage. The carapace length, carapace width, and body weight of each crab were measured prior to tissue collection (Table S1). Fresh hepatopancreas tissue from each crab was quickly collected and immediately stored into liquid nitrogen for RNA isolation.

### RNA isolation and RNA-Seq library preparation

Total RNA was extracted from approximately 80 mg of hepatopancreas tissue with TRIzol Reagent (Invitrogen) according to the manufacturer’s instructions. The RNA integrity and quantity were determined using an Agilent 2100 Bioanalyzer (Agilent, Shanghai, China). A total of 4 μg RNA with RNA integrity number above 7.0 was used for RNA-Seq library construction using the NEBNext® UltraTM RNA Library Prep Kit for Illumina (NEB, USA). Indexed libraries were then pooled and sequenced on Illumina Hiseq^TM^2500, with 150 bp pair-end reads produced.

### Cleaning and assembly of transcriptome sequencing reads

Raw reads from all four stages were first combined and quality-filtered using the Trimmomatic read trimming tool^26^. Reads containing 3′ or 5′ ends with an average quality score below 20 in a 4 bp sliding window were trimmed, and reads with quality below 10 at the beginning or the end were also removed^26^. Any reads shorter than 120 bp were excluded from further assembly. All the cleaned reads were used for the reference transcriptome assembly based on Trinity version 2014-04-13 with the paired-end mode^27^. To filter out the misassembled transcripts, raw sequenced reads were mapped to the assembled reference transcriptome using Bowtie 1.0.0^28^. Transcript abundance was estimated using RSEM software, the FPKM (fragments per kilobase per transcript per million mapped reads) value was calculated, and transcripts with FPKM < 1 were filtered out^29^. Filtered transcripts were then used as *E. sinensis* reference transcriptome for downstream analysis.

To identify transcriptomic differences amongst the four molting stages of the molting cycle (PoM, InM-I, InM-II and PrM), four *de novo* assembled transcriptomes (one at each stage) were assessed using Trinity software. To investigate the accuracy of the assembled transcriptome, raw reads were mapped to the assembled transcriptome using Bwa-0.7.9a with BWA-MEM algorithm^30^, and the mapping statistics was calculated using SAMtools 0.1.19 with the samtools flagstat command^31^. To test the saturation level of our assembled transcriptome, 20, 40, 60, 80, and 100 million reads were randomly selected from the raw reads and then assembled using Trinity software, and the number of transcripts with FPKM ≥ 1 was counted.

### Transcriptome annotation

Assembled transcriptomes were annotated using BLASTX against NCBI-NR and UniProt protein databases, with a cutoff E-value smaller than 1e-6. The BLASTX results were imported into BLAST2GO software, and Gene Ontology (GO) terms and EC numbers for the KEGG pathway were annotated^32^. The protein-coding DNA sequence region (CDS) was predicted using TransDecoder implemented in Trinity software. Sequences with a corresponding protein length greater than 100 were retained for further analysis. The predicted CDS was also subjected to BLASTP against the eggNOG V4.0 database to predict Clusters of Orthologous Groups (COGs)^33^. SignalP 4.1 software was used for predicting the presence and location of signal peptide cleavage sites in amino acid sequences^34^. To predict transcription factors (TFs), protein sequences were used as queries against the PFAM-A database using HMMER v3.1b1 software with hmmscan command^35^. The curated DNA-binding domain HMM list (PFAM 18) download from the DBD database was used to map the TF ID^36^. To identify novel noncoding RNAs, we removed the transcripts that could be annotated as functional genes based on the NCBI-NR database, as well as transcripts with the number of amino acids more than 100. The remaining transcripts were used as query sequences to BLAST against the Rfam database using rfam_scan.pl_v_1.04 software^37^.

### Differential expression analysis

The filtered transcriptome generated through *de novo* assembly was used as a reference transcriptome for RNA-Seq expression analysis. Raw reads generated from each stage were mapped to the reference transcriptome, and FPKM values were calculated by RSEM software^29^. The resulting data matrix that contains the expression value (FPKM) for the samples of all four stages was generated by “rsem-generate-data-matrix” script. This data matrix was imported into edgeR 2.14 to identify the differentially expressed genes (DEGs) with *P *< 0.001 for FDR^38^. FPKM values of DEGs were normalized by log2 and median centered, and cluster analysis was performed using the hierarchical cluster method based on Euclidean distance using Trinity Perl script^27^. We chose clusters of genes that showed different expression patterns following the instructions of Trinity software. For each defined cluster, expression patterns were plotted by R scripts. GO enrichment analysis of the DEGs detected in specific clusters was conducted by DAVID function annotation tools^39^.

### qRT-PCR validation

To validate the results from RNA-Seq differential gene expression analysis, quantitative real-time PCR (qRT-PCR) was carried out amongst four molting stage. Twelve genes from different gene expression clusters were chosen for qRT-PCR assays. PCR primers were designed based on the assembled transcriptome sequences (Table S2). Internal reference genes were chosen based on the estimation of RefFinder (http://www.leonxie.com/referencegene.php). RefFinder integrates the currently available major computational programs (geNorm, Normfinder, and BestKeeper) and the comparative ∆Ct method to compare and rank the tested candidate reference genes^40^,^41^,^42^,^43^. We used *alpha-tubulin*, *ubiquitin conjugating enzyme*, and *β-actin* as internal reference genes to normalize the gene expression level (Figure S1). The qRT-PCR was conducted by using SYBR Green Premix Ex Taq (Takara, Japan) in a CFX96 real-time PCR system (Bio-Rad, USA). A standard curve was firstly generated to assess accuracy, and primers with efficiency of amplification between 95% and 105% were chosen for following qRT-PCR. Four biological and three technical replicates were used for each gene. qRT-PCR was performed in a 25 μl reaction mixture including 12.5 μl SYBR Premix Ex Taq^TM^ II (2×), 1 μl each primer (10 μM), 2 μl cDNA, and 8.5 μl ddH_2_O. The PCR procedure used was: 95 °C for 30 s, followed by 40 cycles of 95 °C for 5 s and 60 °C for 30 s, a 0.5 °C/5 s incremental increase from 60 °C to 95 °C, 30 s elapse time for each cycles. The relative expression was estimated using the 2^–∆∆Ct^ method with PoM stage samples as calibration control, therefore, ∆∆Ct = [(C_t_, _target_ – C_t_, _reference_) _molt stage_] – [(C_t_, _target_ – C_t_, _reference_) _PoM stage_]^44^. Relative expression results were presented as the fold-change relative to PoM stage. Statistical significance (*P *< 0.05) was determined using one-way ANOVA and Duncan’s multiple range tests under SPSS 17.0.

## Results

### Transcriptome assembly

A total of 125,852,483 paired-end reads with 150 bp each were obtained from 12 samples at the four molting stages. After trimming low quality reads, 100,979,293 clean reads were used for *de novo* transcriptome assembly, resulting in 256,361 transcripts (each with more than 200 bp). The N50 for the assembled transcripts were 766 bp and the average length was 585 bp. A large portion of transcripts were smaller than 400 bp (Figure S2A). After filtering transcripts with FPKM < 1, 97,398 transcripts remained for downstream analysis (Figure S3). These transcripts resulted in a filtered reference transcriptome with N50 of 1,432 bp and an average length of 746 bp (Figure S2B). Although the number of transcripts decreased after filtering, the proportion of reads mapped to the transcriptomes was comparable (87.7% vs 86.3%) (Table 1), indicating the transcripts with FPKM < 1 were likely to be misassembled.

**Figure 2:**
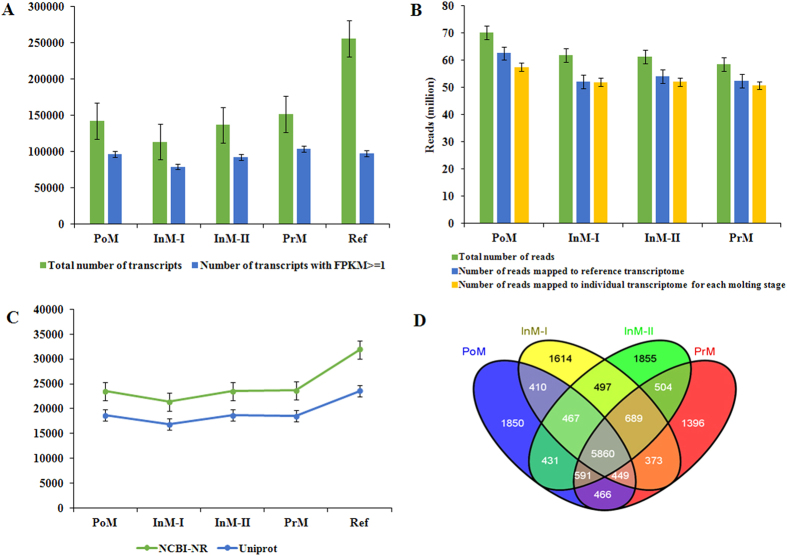
Assembly, mapping, annotation and comparisons of transcriptomes in different molting stages of Chinese mitten crab. (**A**) Total number of *de novo* assembled transcripts and number of transcripts with FPKM ≥ 1; (**B**) Total number of reads, number of reads mapped to reference transcriptome and number of reads mapped to stage-specific transcriptome for each molting stage; (**C**) Number of transcripts annotated using NCBI-NR and UniProt protein database; (**D**) Venn diagram for the number of annotated genes. PoM: postmolt (Day 2), InM-I: intermolt-I (Day 10), InM-II: intermolt-II (Day 20), and PrM: premolt (Day 30).

The number of transcripts assembled from over 60 million reads varied from 113,599 to 151,932, depending on the molting stage. The number of transcripts with FPKM ≥ 1 ranged from 79,273 to 104,104 (Fig. 2A, Table S3). Sequencing reads from the specimens of each stage were mapped back to the filtered reference transcriptome and the corresponding stage-specific transcriptome. More than 80% of the reads were mapped to the filtered reference transcriptome and the corresponding transcriptome (Fig. 2B, Table S4). The number of transcripts (FPKM ≥ 1) assembled from 20, 40, 60, 80, and 100 million clean reads ranged from 82,379 to 102,527, indicating that 20 million clean reads were nearly sufficient for *E. sinensis* transcriptome assembly. These results demonstrated that our reference transcriptome assembled with more than 100 million clean reads was saturated and thus of very high quality (Figure S4).

### Transcriptome annotation

BLASTX searches against two protein databases (NCBI-NR and UniProt) resulted in 31,900 (32.8% of total transcripts) and 23,582 transcripts annotated, respectively, with significant BLAST hits to the reference transcriptome (Fig. 2C, Table 1). Most top BLAST hits were matched to *Daphnia pulex* sequences (Figure S5). Further GO analysis showed that 24,402 transcripts were mapped to at least one GO category (Figure S6). In biological processes, most of the GO-mapped transcripts were related to cellular and metabolic processes. A high number of transcripts (2,280) were mapped to the GO category: response to stimulus (GO:0050896). In addition, we identified 91, 170, and 753 transcripts involved in growth (GO:0040007), reproduction (GO:0000003), and developmental processes (GO:0032502), respectively. A total of 14,760 transcripts were annotated with corresponding enzymes, and 131 KEGG pathways were identified through functional analysis of the *E. sinensis* transcriptome (Table S5). Purine metabolism, pyrimidine metabolism, thiamine metabolism, pyruvate metabolism, and oxidative phosphorylation were the top pathways with the highest number of genes represented.

After TransDecoder prediction, the number of predicted protein coding sequences (CDS) with a minimum amino acid length of 100 was 36,948. The predicted protein sequences were used for BLASTP against the eggNOG database to find COGs, and the results are presented in Figure S7. A total of 28,904 transcripts were found in one COG group. Most proteins were annotated as “S: functional unknown” due to the lack of annotated protein sequences of closely related species. Using predicted amino acid sequences, 2,149 signal peptides with cleavage sites were predicted (Table S6). With the HMMER software and PFAM-A database, 1,167 protein sequences were predicted to have 106 TFs (Table S7) compared with 112 TFs in *D. pulex*^36^. The top 20 TFs in *E. sinensis* were compared with the top 20 TFs in *D. pulex* (Figure S8A,B), and both had around 60% C2H2-type zinc finger (PF00096) TFs. In addition, we were able to identify 135 transcripts corresponding to 27 noncoding RNAs that include micro-RNAs, small RNAs, 5S_rRNAs, and tRNAs (Table S8).

The number of transcripts annotated through BLAST search against NCBI-NR database ranged from 21,405 to 23,666, depending on the molting stage, and the number of transcripts annotated with UniProt ranged from 16,881 to 18,715 (Fig. 2C). We identified 5,860 annotated genes shared among stages (Fig. 2D) corresponding to 18,014, 16,509, 17,782, and 18,032 transcripts in PoM, InM-I, InM-II, and PrM, respectively. The numbers of transcripts in the PrM and PoM stages were significantly higher than those in the InM-I and InM-II stages (*P *< 0.01, t-test) (Table S9).

### Differentially expressed genes (DEGs) in different molting stages

**Figure 3:**
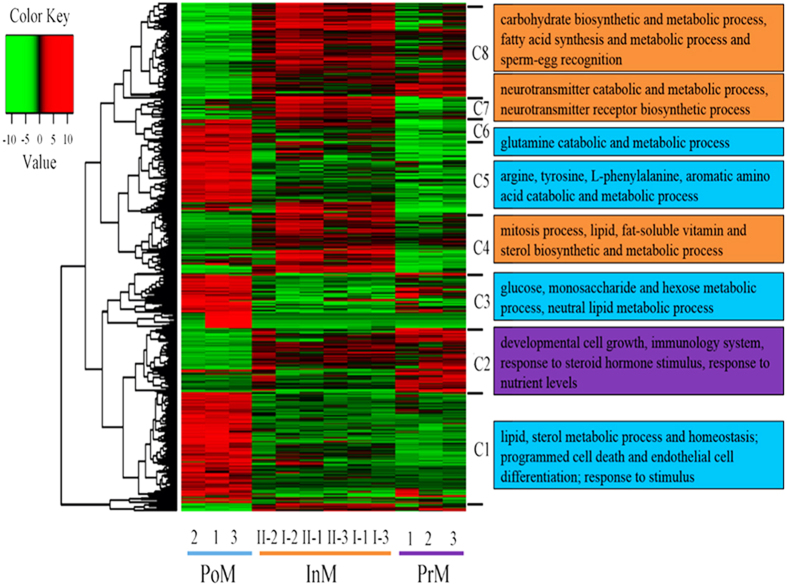
Heat map of differentially expressed genes clustered in two ways - molting stages and GO categories. PoM: postmolt (Day 2), InM-I: intermolt-I (Day 10), InM-II: intermolt-II (Day 20), and PrM: premolt (Day 30). Numbers - replicate samples, C1–C8 - functional clusters of differentially expressed genes, and Color key value - FPKM fold change.

A total of 1,189 differentially expressed genes (DEGs) were identified across the four molting stages. No DEGs were observed between the two intermolt stages (InM-I and InM-II); more DEGs were found in PoM compared with other stages (Fig. 3). We identified 740, 491, and 550 genes differentially expressed respectively between PoM and each of other three stages, InM-I, InM-II and PrM. A total of 195 DEGs were identified between PrM and InM-I whereas 24 DEGs were found between PrM and InM-II. Eight clusters were defined based on the hierarchical clustering results revealing different expression patterns in the four stages (Fig. 3 and S9). DEGs in clusters 1, 3, 5, and 6 were highly expressed in PoM compared to other stages. DEGs in cluster 2 showed that the expression of genes increased with the days following molting (from PoM to PrM). Clusters 4, 7, and 8 showed that the genes had low expression levels in the PoM and PrM stages but relatively high expression levels in the InM-I and InM-II stages (Fig. 3 and S9).

GO enrichment analysis of differentially expressed genes showed that genes in clusters 1, 3, 5, and 6 (highly expressed in PoM; Fig. 3) were mainly associated with “energy consumption process”, “homeostasis process”, and “response to stimulus”. Genes such as *sterol o-acyltransferase 1*(*soat1*), *niemann-pick C1*(*npc1*), *apoptosis 2 inhibitor* (*iap2*)*, G-protein coupled receptor moody* (*moody*), and *nocturnin* (*ccrn4l*) in cluster 1 were enriched in biological processes such as lipid and sterol homeostasis and metabolism, programmed cell death and endothelial cell differentiation, response to stimulus (smell, chemicals, and cocaine), and rhythmic processes (Fig. 3, Table S10). Genes such as *glucose-6-phosphate translocase* (*g6pt1*), *carnitine O-palmitoyltransferase 1* (*cpt1a*), *phosphoenolpyruvate carboxykinase* (*pck*), *fumarylacetoacetase* (*fah*), *long chain acyl-CoA synthetase 8* (*lacs8*), *probable proline dehydrogenase 2* (*prodh2*), and *glutamate dehydrogenase* (*gdh*) in clusters 3, 5, and 6 were associated with glucose, monosaccharide, and hexose metabolism; neutral lipid metabolism, and fatty-acid metabolism; and amino acid catabolic and metabolic processes (Fig. 3, Table S10).

DEGs in clusters 4, 7, and 8 (highly expressed in InM-I and InM-II) were related to energy storage and specific developmental processes. Genes such as *probable C-5 sterol desaturase* (*erg3*), *aldehyde dehydrogenase family 8 member A1* (*aldh8a1*), *fatty acid synthase (fas)*, *ATP-citrate synthase* (*acly*), *mitotic checkpoint serine* (*bub1*), *centromere protein F* (*cenpf*), *actin-binding protein anillin* (*anln*) and *cyclin-A2* (*ccna2*) in cluster 4 were strongly enriched in lipids, fat-soluble vitamins, cholesterol and sterol biosynthetic process, and mitosis; they were mostly related to the accumulation of nutrient substances and cell proliferation (Fig. 3, Table S10). Genes such as *aldehyde dehydrogenase* (*aldh2*), *superoxide dismutase* (*sod2*), and *acetylcholinesterase* (*ache*) in cluster 7 were enriched in liver development, neurotransmitter receptor biosynthetic process, receptor biosynthetic process, and neurotransmitter catabolic and metabolic processes. Genes such as *glucose-6-phosphate isomerase* (*pgi*), *triosephosphate isomerase B* (*tpi1b*), *phosphoglycerate mutase 2* (*pgam2*), *ATP-dependent 6-phosphofructokinase* (*pfk*), *phosphoglycerate kinase* (*pgk*), *ADP-dependent glucokinase* (*adpgk*), *elongation of very long chain fatty acids protein 6* (*elovl6*), and *acyl-coA desaturase (scd)* in cluster 8 were strongly enriched in carbohydrate metabolic, biosynthesis, fatty acid synthesis and metabolism (Fig. 3, Table S10).

The DEGs in cluster 2 (increase form PoM to PrM) were enriched for genes *broad-complex core protein isoforms 1(br)*, *ecdysone receptor (ecr)*, *niemann-pick C1* (*npc1*), *arylsulfatase B* (*arsb*), and *forkhead box protein P1* (*foxp1*) associated with the response to steroid hormone stimulus, nutrient levels and acid transport, developmental cell growth, RNA transcription, and immune system development (Fig. 3, Table S10).

### qRT-PCR validation

**Figure 4:**
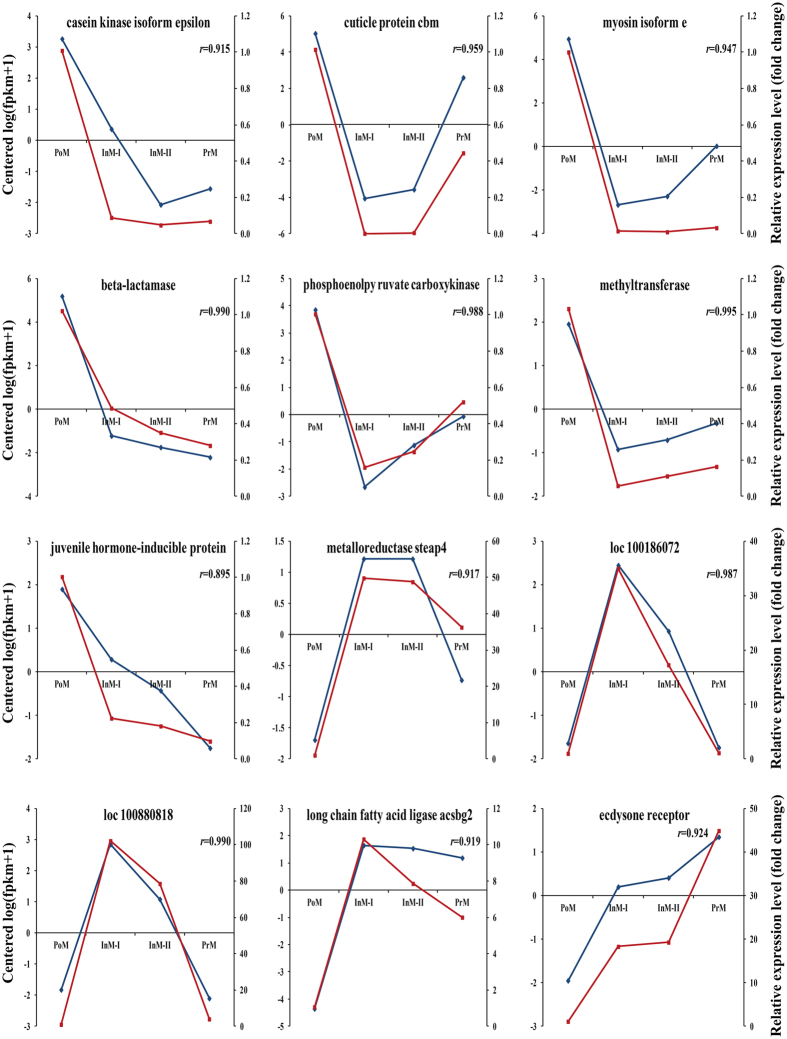
Expression profiles of 12 differentially expressed genes from RNA-Seq (blue) and qRT-PCR (red) with *alpha-tubulin* as reference gene in different molting stages.

Twelve genes were selected from the clusters of differentially expressed genes and then verified by qRT-PCR. One gene (*casein kinase isoform epsilon*) from cluster 1, four genes (*cuticle protein cbm*, *myosin isoform e*, *beta-lactamase*, and *phosphoenolpyruvate carboxykinase*) from cluster 3 and two genes (*methyltransferase* and *juvenile hormone-inducible protein*) from cluster 5 had the highest expression levels during postmolt. Three genes (*metalloreductase steap4*, *loc100186072* and *loc100880818*) from cluster 4, one gene (*long chain fatty acid ligase acsbg 2*) from cluster 8 presented the highest expression levels in intermolt. One gene (*ecdysone receptor*) from cluster 2 showed the highest expression level in premolt. qRT-PCR efficiency of amplification was above 95% and the correlation value (*R*^2^) was more than 0.98 for each gene (Table S2). The qRT-PCR results confirmed the findings of RNA-Seq (Fig. 4 and S10), with a correlation coefficient (*r*) from 0.798 to 0.997 depending on specific genes and reference genes.

## Discussion

### Transcriptomic repertoire for Chinese mitten crab

This study established a comprehensive repertoire of annotated genes, differentially expressed genes, non-coding RNA, and transcription factors for the Chinese mitten crab from different molting stages throughout one molting cycle. In order to produce a comprehensive reference transcriptome, all raw reads from the four different molting stages were combined (more than 120 million paired-end reads) and assembled. The saturation analysis and the comparison of transcription factors between *E. sinensis* and *Daphnia pulex* (water flea) asserted a high-quality hepatopancreas transcriptome for *E. sinensis*.

Gene Ontology functional annotation analysis revealed a relatively large number of transcripts in “response to stimulus,” which suggest these genes may be involved in the adjustment of permeation pressure during the transition from saltwater to freshwater during the crab’s lifecycle and presumably prevent automutilation^45^. KEGG analysis demonstrated the top enriched pathways are energy related ones, including oxidative phosphorylation and glycolysis/gluconeogenesis. This is consistent with the assumption that the molting process requires a significant amount of energy to uptake water and swell the exoskeleton and to produce chitin.

Crustaceans are the second largest group of arthropods, the most speciose and morphologically diverse animal group on the planet^46^. To date, there is only one crustacean species - *Daphnia pulex* (water flea) with its genome sequence publically available, although a number of transcriptomic studies have been reported to address issues such as salinity stress, immune responses to microbial pathogens, and the development of sexual organs in *E. sinensis*^47^,^48^,^49^,^50^. As of May 28, 2015, there are 9 Bio-Samples with over 60 Gb sequence data available in *E. sinensis* in the NCBI SRA database. One future direction for this project is to sequence transcriptomes of other tissues to corroborate the preliminary findings in this study and to integrate publically available transcriptomic data to enhance our transcriptomic repertoire for the Chinese mitten crab. Nevertheless, our transcriptomic data and analysis results will facilitate genomic sequencing and annotation of other crustaceans (including the Chinese mitten crab) in the future.

### Transcriptomic variation among different molting stages

A comparable number of annotated transcripts were attained for each of the four transcriptomes assembled from different molting stages (Fig. 2C), despite the difference in the number of total assembled transcripts in different stages (Fig. 2A). The largest numbers of assembled transcripts were found in stages postmolt (PoM) and premolt (PrM), which are the two critical stages that may have more developmental and immune response genes involved as discussed below. With the mapping of raw reads from each molting stage to the reference transcriptome, we successfully identified differentially expressed genes and gene clusters associated with energy metabolism and physiological responses during one molting cycle in the *E. sinensis* hepatopancreas.

Postmolt (PoM) is a period when the Chinese mitten crab is recovering from molting. During postmolt the exoskeleton of *E. sinensis* is quickly hardened as a mechanism to avoid predation, which requires sufficient energy input. Our clustering analysis revealed several clusters of highly expressed genes during the postmolt stage. The genes in cluster 1 are involved in homeostasis (*soat1*, *npc1*), response to stimulus, and programmed cell death and differentiation, which may suggest the crabs under postmolt are very sensitive to the external and internal environments. Clusters 3, 5 and 6 contain highly expressed genes enriched in carbohydrate, neutral lipid, and amino acid metabolic process (pck, *g6pt1*, *Cpt1a*, *Fah*, and *prodh2*), which may provide more energy to maintain homeostasis when dietary carbohydrate is unavailable in the PoM stage (Table S10, cluster 3, 5, 6)^51^,^52^,^53^,^54^. The *pck* gene has been considered as a vital marker for liver gluconeogenesis - a metabolic pathway to produce glucose when starvation occurs for extended periods^51^,^52^. Furthermore, we found the cuticle protein *cbm* gene (likely involved in chitin metabolic process) enriched in cluster 3. The over expression of this gene is likely to promote the synthesis of chitin to quickly harden their shell for defense.

Intermolt (InM-I and InM-II) is an important period in growth and development and thus it is an essential stage for energy storage^55^,^56^. We found a number of highly expressed genes associated with the glycolysis pathway. These genes including *adpgk*, *pgi*, *pfk*, *tpi1b*, *pgk* and *pgam2*, are involved in the conversion of glucose into pyruvic acid to produce energy. Note that the two genes, *adpgk* and *pfk,* are associated with the irreversible steps in this glycolysis process compared to gluconeogenesis^52^. The product of glycolysis, i.e., pyruvic acid, could supply sufficient energy to cells through the citric acid cycle under aerobic condition and consequently facilitate the growth and development for *E. sinensis* in InM stage. Our clustering analysis revealed several highly expressed genes such as *bub1*, *cenpf*, *anln* and *ccna2*, and these genes are likely pertaining to mitosis (Table S10, cluster 4). Two genes, *aldh2* and *sod2,* were linked to liver development, and one gene, *ache,* was presumably related to eye development (Table S10, cluster 7).

During intermolt (InM-I and InM-II) fatty acid synthesis occurs and it is primarily controlled by the *fas* and *acly* genes, which catalyze the lipids synthesis pathway through converting the carbohydrates into fatty acids^18^,^57^,^58^. We found these two genes highly expressed in InM stage in addition to the genes involved in lipid synthesis such as *erg3*, *aldh8a1*, *elovl6*, and *scd*. These genes are likely to convert excessive carbohydrates into fatty acids that could be eventually stored in the hepatopancreas^52^. The storing of lipids shall play an important role in energy supply when *E. sinensis* is fasting in other molting stages^12^. Several studies have reported that crustaceans synthesize a large amount of carbohydrates, lipids, fatty acids, and fat-soluble vitamins during the intermolt stage for subsequent molting and limb regeneration^59^. Reduction in food supply has been linked to a longer intermolt period^59^.

During premolt, we found the ecdysone receptor gene differentially expressed. This gene is an essential regulatory element for ecdysone hormone and has been shown to regulate the molting of crustaceans^6^,^60^. Other enriched genes included those associated with steroid hormone stimulus, immune system, response to nutrient levels, and amino acid transport. Gene expression analysis revealed several other genes highly expressed during the premolt (PrM) stage, including *EcR*, *br* and *foxp1. EcR* is a heterodimer composed of the products of the *EcR* and *RXR/USP* nuclear hormone genes and was found to regulate the ecdysone hormone, one vital hormone for successful molting in crustaceans^9^,^61^,^62^,^63^,^64^. Up-regulation of *EcR* has been observed in other crustacean species such as the freshwater prawn *Macrobrachium nipponense*, Pacific white shrimp, *Litopenaeus vannamei*, and grapsid crab*, Metopograpsus messor* during the premolt stage^65^,^66^,^67^. The *br* and *foxp1* genes are assumed to be associated with responses to steroid hormone stimuli as shown in the fruit fly, *Drosophila melanogaster,* and honeybee, *Apis mellifera*^68^,^69^.

Understanding the dynamic process of molting is an essential goal for both aquaculture and invasive species management. For aquaculture, the intermolt stage is an ideal period to enhance nutrition to shorten the intermolt period interval and foster growth of crabs. During the postmolt stage it is essential to increase protection for these crabs in the form of additional habitats to ensure they are not victims of predation. For invasive species management, limiting the amount of nutrients available to the crabs is essential to extend the intermolt period in order to limit the number of sexually mature individuals. Furthermore, additional research needs to be done to determine if the postmolt stage might be extended which would cause invasive crabs to be more susceptible to predation as potential management technique.

In conclusion, we performed a comprehensive transcriptomic study on the hepatopancreas of *E. sinensis* amongst four molting stages. We established the first reference transcriptome of the hepatopancreas of *E. sinensis*, characterized genes and functional categories at each molting stage, and identified regular patterns of differentially expressed genes amongst different molting stages. Our study thus provides unique insight into the functions of the hepatopancreas in energy metabolism and biological processes for molting in crustaceans. Future research directions include transcriptomic studies of more tissues and samples from different molting cycles and genomic sequencing of the Chinese mitten crab.

### Data Archiving

Sequencing reads are available at NCBI SRA database (SRX824594, SRX845724, and SRX845725).

## Additional Information

**How to cite this article**: Huang, S. *et al.* Transcriptomic variation of hepatopancreas reveals the energy metabolism and biological processes associated with molting in Chinese mitten crab, *Eriocheir sinensis*. *Sci. Rep.* **5**, 14015; doi: 10.1038/srep14015 (2015).

## Supplementary Material

Supplementary Information

Supplementary Dataset 1

Supplementary Dataset 2

## Figures and Tables

**Table 1:** Summary statistics of Chinese mitten crab transcriptome.

**Sequencing**		
Raw reads (pair-end)	125,852,483	
Clean reads (pair-end)	100,979,293	
Total nucleotides (bp)	3.8 × 10^10^	
Assembly		
	*De novo* assembled	Filtered (FPKM ≥ 1)*
No. of transcripts	256,361	97,398
No. of N50 transcripts	41,705	13,081
N50 length	766	1,432
Mean transcripts length	585	746
Largest transcripts	32,421	32,421
Reads Mapped	87.7%	86.3%
Annotation		
NCBI-Nr	31,900	
UniProt	23,582	
GO	24,402	
KEGG	14,760	
COG	28,904	

^*^FPKM: fragments per kilobase per transcript per million mapped reads.

## References

[b1] Panganiban G., Sebring A., Nagy L. & Carroll S. The development of crustacean limbs and the evolution of arthropods. Science. 270, 1363–1366 (1995). 7481825 10.1126/science.270.5240.1363

[b2] Hopkins P. M. Limb Regeneration in the Fiddler Crab, *Uca pugilator*: Hormonal and Growth Factor Control. Am Zool. 41, 389–398 (2001).

[b3] Morris S. *et al.* The adaptive significance of crustacean hyperglycaemic hormone (CHH) in daily and seasonal migratory activities of the Christmas Island red crab *Gecarcoidea natalis*. J Exp Biol. 213, 3062–3073 (2010). 20709934 10.1242/jeb.045153

[b4] Jung H., Lyons R. E., Hurwood D. A. & Mather P. B. Genes and growth performance in crustacean species: a review of relevant genomic studies in crustaceans and other taxa. Rev Aquacult. 5, 77–110 (2013).

[b5] Kurup N. The intermolt cycle of an anomuran, Petrolisthes cinctipes Randall (Crustacea-Decapoda). Biol Bull. 127, 96–107 (1964).

[b6] Chang E. S. & Mykles D. L. Regulation of crustacean molting: A review and our perspectives. Gen Comp Endocr. 172, 323–330 (2011). 21501612 10.1016/j.ygcen.2011.04.003

[b7] Hopkins P. Crustacean Ecdysteriods and Their Receptors in Ecdysone: Structures and Functions (ed Smagghe Guy) Ch. 3, 73–97 (Springer Netherlands, 2009).

[b8] Chen H.-Y., Dillaman R. M., Roer R. D. & Watson R. D. Stage-specific changes in calcium concentration in crustacean (*Callinectes sapidus*) Y-organs during a natural molting cycle, and their relation to the hemolymphatic ecdysteroid titer. Comp Biochem Phys A. 163, 170–173 (2012). 10.1016/j.cbpa.2012.05.205 22683690

[b9] Nakagawa Y. & Henrich V. C. Arthropod nuclear receptors and their role in molting. FEBS J. 276, 6128–6157 (2009). 19796154 10.1111/j.1742-4658.2009.07347.x

[b10] Wang C., Li C. & Li S. Mitochondrial DNA-inferred population structure and demographic history of the mitten crab (*Eriocheir sensu stricto*) found along the coast of mainland China. Mol Ecol. 17, 3515–3527 (2008). 19160479 10.1111/j.1365-294x.2008.03850.x

[b11] Dittel A. I. & Epifanio C. E. Invasion biology of the Chinese mitten crab *Eriochier sinensis*: A brief review. J Exp Mar Biol Ecol. 374, 79–92 (2009).

[b12] Wang W., Wang C. & Ma X. Ecological Aquaculture of Chinese mitten crab (In Chinese). 2 edn, (China Agriculture Press, 2013).

[b13] Leersnyder M. d., Dhainaut A. & Porcheron P. La vitellogenese chez le crabe *Eriocheir sinensis*. Bull. Soc. zool. Fr. 105, 413–419 (1980).

[b14] Tian Z., Kang X. & Mu S. The molt stages and the hepatopancreas contents of lipids, glycogen and selected inorganic elements during the molt cycle of the Chinese mitten crab *Eriocheir sinensis*. Fish Sci. 78, 67–74 (2012).

[b15] Guo Y.-R., Gu S.-Q., Wang X.-C., Zhao L.-M. & Zheng J.-Y. Comparison of fatty acid and amino acid profiles of steamed Chinese mitten crab. Fish Sci. 80, 621–633 (2014).

[b16] Wang L., Yan B., Liu N., Li Y. & Wang Q. Effects of cadmium on glutathione synthesis in hepatopancreas of freshwater crab, Sinopotamon yangtsekiense. Chemosphere. 74, 51–56 (2008). 18952253 10.1016/j.chemosphere.2008.09.025

[b17] Uawisetwathana U. *et al.* Insights into eyestalk ablation mechanism to induce ovarian maturation in the black tiger shrimp. PloS one. 6, e24427 (2011). 21915325 10.1371/journal.pone.0024427 PMC3168472

[b18] Wang W., Wu X., Liu Z., Zheng H. & Cheng Y. Insights into Hepatopancreatic Functions for Nutrition Metabolism and Ovarian Development in the Crab *Portunus trituberculatus*: Gene Discovery in the Comparative Transcriptome of Different Hepatopancreas Stages. PLoS One. 9, e84921 (2014). 24454766 10.1371/journal.pone.0084921 PMC3890295

[b19] Jiang H., Yin Y., Zhang X., Hu S. & Wang Q. Chasing relationships between nutrition and reproduction: A comparative transcriptome analysis of hepatopancreas and testis from *Eriocheir sinensis*. Comp Biochem Phys D. 4, 227–234 (2009). 10.1016/j.cbd.2009.05.001 20403758

[b20] Mykles D. L. Ecdysteroid metabolism in crustaceans. J Steroid Biochem. 127, 196–203 (2011). 10.1016/j.jsbmb.2010.09.001 20837145

[b21] Wang Z., Gerstein M. & Snyder M. RNA-Seq: a revolutionary tool for transcriptomics. Nat Rev Genet. 10, 57–63 (2009). 19015660 10.1038/nrg2484 PMC2949280

[b22] Wachholtz M. *et al.* Transcriptome analysis of two buffalograss cultivars. BMC Genomics. 14, 613 (2013). 24024986 10.1186/1471-2164-14-613 PMC3846939

[b23] Wang C., Wachholtz M., Wang J., Liao X. & Lu G. Analysis of the skin transcriptome in two oujiang color varieties of common carp. PloS one. 9, e90074 (2014). 24603653 10.1371/journal.pone.0090074 PMC3946065

[b24] He J. Research on the growth of the cultured population of Chinese mitten crab (In Chinese). Reser Fisher. 25, 10–11 (2005).

[b25] Phlippen M. K., Webster S. G., Chung J. S. & Dircksen A. H. Ecdysis of decapod crustaceans is associated with a dramatic release of crustacean cardioactive peptide into the haemolymph. J Exp Biol. 203, 521–536 (2000). 10637181 10.1242/jeb.203.3.521

[b26] Bolger A. M., Lohse M. & Usadel B. Trimmomatic: A flexible trimmer for Illumina Sequence Data. Bioinformatics. 1–7 (2014). 10.1093/bioinformatics/btu170 PMC4103590 24695404

[b27] Grabherr M. G. *et al.* Full-length transcriptome assembly from RNA-Seq data without a reference genome. Nat Biotech. 29, 644–652 (2011). 10.1038/nbt.1883 PMC3571712 21572440

[b28] Langmead B., Trapnell C., Pop M. & Salzberg S. Ultrafast and memory-efficient alignment of short DNA sequences to the human genome. Genome Biol. 10, 1–6 (2009). 10.1186/gb-2009-10-3-r25 PMC2690996 19261174

[b29] Li B. & Dewey C. RSEM: accurate transcript quantification from RNA-Seq data with or without a reference genome. BMC Bioinformatics. 12, 323 (2011). 21816040 10.1186/1471-2105-12-323 PMC3163565

[b30] Li H. & Durbin R. Fast and accurate long-read alignment with Burrows–Wheeler transform. Bioinformatics. 26, 589–595 (2010). 20080505 10.1093/bioinformatics/btp698 PMC2828108

[b31] Li H. *et al.* The Sequence Alignment/Map format and SAMtools. Bioinformatics. 25, 2078–2079 (2009). 19505943 10.1093/bioinformatics/btp352 PMC2723002

[b32] Conesa A. *et al.* Blast2GO: a universal tool for annotation, visualization and analysis in functional genomics research. Bioinformatics. 21, 3674–3676 (2005). 16081474 10.1093/bioinformatics/bti610

[b33] Powell S. *et al.* eggNOG v4.0: nested orthology inference across 3686 organisms. Nucleic Acids Res. 42, 231–239 (2013). 10.1093/nar/gkt1253 PMC3964997 24297252

[b34] Petersen T. N., Brunak S., von Heijne G. & Nielsen H. SignalP 4.0: discriminating signal peptides from transmembrane regions. Nat Meth. 8, 785–786 (2011). 10.1038/nmeth.1701 21959131

[b35] Finn R. D., Clements J. & Eddy S. R. HMMER web server: interactive sequence similarity searching. Nucleic Acids Res. 39, 29–37 (2011). 10.1093/nar/gkr367 PMC3125773 21593126

[b36] Wilson D., Charoensawan V., Kummerfeld S. K. & Teichmann S. A. DBD—taxonomically broad transcription factor predictions: new content and functionality. Nucleic Acids Res. 36, 88–92 (2008). 10.1093/nar/gkm964 PMC2238844 18073188

[b37] Burge S. W. *et al.* Rfam 11.0: 10 years of RNA families. Nucleic Acids Res. 41, 226–232 (2012). 10.1093/nar/gks1005 PMC3531072 23125362

[b38] Robinson M. D., McCarthy D. J. & Smyth G. K. edgeR: a Bioconductor package for differential expression analysis of digital gene expression data. Bioinformatics. 26, 139–140 (2010). 19910308 10.1093/bioinformatics/btp616 PMC2796818

[b39] Huang D. W., Sherman B. T. & Lempicki R. A. Systematic and integrative analysis of large gene lists using DAVID bioinformatics resources. Nat Protocols. 4, 44–57 (2008). 10.1038/nprot.2008.211 19131956

[b40] Vandesompele J. *et al.* Accurate normalization of real-time quantitative RT-PCR data by geometric averaging of multiple internal control genes. Genome Biol. 3, 1–12 (2002). 10.1186/gb-2002-3-7-research0034 PMC126239 12184808

[b41] Andersen C. L., Jensen J. L. & Ørntoft T. F. Normalization of Real-Time Quantitative Reverse Transcription-PCR Data: A Model-Based Variance Estimation Approach to Identify Genes Suited for Normalization, Applied to Bladder and Colon Cancer Data Sets. Cancer Research. 64, 5245–5250 (2004). 15289330 10.1158/0008-5472.CAN-04-0496

[b42] Pfaffl M., Tichopad A., Prgomet C. & Neuvians T. Determination of stable housekeeping genes, differentially regulated target genes and sample integrity: BestKeeper – Excel-based tool using pair-wise correlations. Biotechnol Lett. 26, 509–515 (2004). 15127793 10.1023/b:bile.0000019559.84305.47

[b43] Silver N., Best S., Jiang J. & Thein S. Selection of housekeeping genes for gene expression studies in human reticulocytes using real-time PCR. BMC Mol Biol. 7, 33 (2006). 17026756 10.1186/1471-2199-7-33 PMC1609175

[b44] Livak K. J. & Schmittgen T. D. Analysis of relative gene expression data using real-time quantitative PCR and the 2(-Delta Delta C(T)) Method. Methods. 25, 402–408 (2001). 11846609 10.1006/meth.2001.1262

[b45] Mao H., Huang S., Wang Z., Zhou L. & Wang C. Molecular Cloning and Expression Analysis of Na+/K+-ATPase α1 Gene in Chinese Mitten Crab (*Eriocheir sinensis*) (In Chinese). J Agricult Biotech. 22, 343–350 (2014).

[b46] Brusca R. & Brusca G. Invertebrates. 2 edn, (Sinauer Associates, Sunderland, MA, 2003).

[b47] Colbourne J. K. *et al.* The Ecoresponsive Genome of *Daphnia pulex*. Science. 331, 555–561 (2011). 21292972 10.1126/science.1197761 PMC3529199

[b48] He L. *et al.* Comparative transcriptome analysis of the accessory sex gland and testis from the Chinese mitten crab (*Eriocheir sinensis*). PLoS One. 8, e53915 (2013). 23342039 10.1371/journal.pone.0053915 PMC3547057

[b49] Li E. *et al.* Transcriptome sequencing revealed the genes and pathways involved in salinity stress of Chinese mitten crab. Eriocheir sinensis. Physiol Genomics. 46, 177–190 (2014). 24423969 10.1152/physiolgenomics.00191.2013

[b50] Cui Z. *et al.* High-density linkage mapping aided by transcriptomics documents ZW sex determination system in the Chinese mitten crab *Eriocheir sinensis*. Heredity. 115, 206–215 (2015). 25873149 10.1038/hdy.2015.26 PMC4814232

[b51] Burgess S. C. *et al.* Cytosolic Phosphoenolpyruvate Carboxykinase Does Not Solely Control the Rate of Hepatic Gluconeogenesis in the Intact Mouse Liver. Cell Metab. 5, 313–320 (2007). 17403375 10.1016/j.cmet.2007.03.004 PMC2680089

[b52] Fromm H. J. & Hargrove M. Essentials of biochemistry. (Springer Science & Business Media, 2012).

[b53] Charkoudian L. K., Farrell B. P. & Khosla C. Natural product inhibitors of glucose-6-phosphate translocase. MedChemComm. 3, 926–931 (2012).

[b54] Jogl G. & Tong L. Crystal Structure of Carnitine Acetyltransferase and Implications for the Catalytic Mechanism and Fatty Acid Transport. Cell. 112, 113–122 (2003). 12526798 10.1016/s0092-8674(02)01228-x

[b55] Devaraj H. & Natarajan A. Molecular mechanisms regulating molting in a crustacean. FEBS J. 273, 839–846 (2006). 16441669 10.1111/j.1742-4658.2006.05117.x

[b56] Kuballa A. & Elizur A. Novel molecular approach to study moulting in crustaceans. Bulletin Fisheries Research Agency Japan. 20, 53 (2007).

[b57] Chirala S. & Wakil S. Structure and function of animal fatty acid synthase. Lipids. 39, 1045–1053 (2004). 15726818 10.1007/s11745-004-1329-9

[b58] Mashima T., Seimiya H. & Tsuruo T. *De novo* fatty-acid synthesis and related pathways as molecular targets for cancer therapy. Brit J cancer. 100, 1369–1372 (2009). 19352381 10.1038/sj.bjc.6605007 PMC2694429

[b59] Hartnoll R. Growth in Crustacea – twenty years on. Hydrobiologia. 449, 111–122 (2001).

[b60] Chang E. S. & Bruce M. J. Ecdysteroid titers of juvenile lobsters following molt induction. J Exp Zool Part A. 214, 157–160 (1980).

[b61] Chang E. S. & O’Connor J. D. Secretion of alpha-ecdysone by crab Y-organs *in vitro*. P Natl Acad Sci USA. 74, 615–618 (1977). 10.1073/pnas.74.2.615 PMC392342 265527

[b62] Keller R. & Schmid E. *In vitro* secretion of ecdysteroids by Y-organs and lack of secretion by mandibular organs of the crayfish following molt induction. J Comp Physiol A. 130, 347–353 (1979).

[b63] Schoettker P. J. & Gist D. H. *In vitro* ecdysteroid production by Y-organs of the blue crab *Callinectes sapidus*. J Crustacean Biol. 10, 487–491 (1990).

[b64] Aranda A. & Pascual A. Nuclear Hormone Receptors and Gene Expression. Physiol Rev. 81, 1269–1304 (2001). 11427696 10.1152/physrev.2001.81.3.1269

[b65] Shen H., Zhou X., Bai A., Ren X. & Zhang Y. Ecdysone receptor gene from the freshwater prawn *Macrobrachium nipponense*: identification of different splice variants and sexually dimorphic expression, fluctuation of expression in the molt cycle and effect of eyestalk ablation. Gen Comp Endocr. 193, 86–94 (2013). 23899714 10.1016/j.ygcen.2013.07.014

[b66] Qian Z. *et al.* Identification of ecdysteroid signaling late-response genes from different tissues of the Pacific white shrimp, *Litopenaeus vannamei*. Comp Biochem Phys A. 172, 10–30 (2014). 10.1016/j.cbpa.2014.02.011 24556071

[b67] Shyamal S., Anilkumar G., Bhaskaran R., Doss G. P. & Durica D. S. Significant fluctuations in ecdysteroid receptor gene (EcR) expression in relation to seasons of molt and reproduction in the grapsid crab, *Metopograpsus messor* (Brachyura: Decapoda). Gen Comp Endocr. 211, 39–51 (2015). 25448252 10.1016/j.ygcen.2014.11.006

[b68] Uhlirova M. *et al.* Use of Sindbis virus-mediated RNA interference to demonstrate a conserved role of Broad-Complex in insect metamorphosis. P Natl Acad Sci USA. 100, 15607–15612 (2003). 10.1073/pnas.2136837100 PMC307615 14668449

[b69] Paul R. K., Takeuchi H. & Kubo T. Expression of Two Ecdysteroid-Regulated Genes, Broad-Complex and E75, in the Brain and Ovary of the Honeybee (*Apis mellifera* L.). Zool Sci. 23, 1085–1092 (2006). 17261922 10.2108/zsj.23.1085

