# Supplementary material for: Transcriptomic variation of hepatopancreas reveals the energy metabolism and biological processes associated with molting in Chinese mitten crab, Eriocheir sinensis
Source: Sci Rep. 2015 Sep 15;5:14015. doi: 10.1038/srep14015 (PMC4570184; doi:10.1038/srep14015)
Supplement: Supplementary Information [file srep14015-s1.pdf]

Supplementary files

Transcriptomic variation of hepatopancreas reveals the energy metabolism and biological processes associated with molting in Chinese mitten crab, *Eriocheir sinensis*

Shu Huang[1$], Jun Wang[1$], Wucheng Yue[1], Jiao Chen[1], Sarah Gaughan[2], Weiqun Lu[1] Guoqing Lu[2*], Chenghui Wang[1*]

[1]Key Laboratory of Freshwater Fisheries Germplasm Resources, Ministry of Agriculture, Shanghai Ocean University, Shanghai, 201306, China

[2]Department of Biology, University of Nebraska at Omaha, Omaha, NE 68182, USA

[$]These authors contributed equally to this work.

*Author for correspondence: Chenghui Wang Email: wangch@shou.edu.cn

Guoqing Lu Email: glu3@unomaha.edu

Supplementary figure legends

Figure S1. Reference genes chosen based on the results of RefFinder.

Figure S2. Length distribution of transcripts of assembled reference transcriptome (A) and filtered reference transcriptome (FPKM≥1) (B).

Figure S3. Number of transcripts with different FPKM values in the assembled reference transcriptome.

Figure S4. Number of transcripts with FPKM ≥1 of the assembled transcriptome from randomly 20 million, 40 million, 60 million, 80 million, and 100 million reads.

Figure S5. Species distribution of top BLAST hits of the transcripts against NCBI NR database.

Figure S6. Functional classification of *E.sinensis* hepatopancreas transcriptome in three Gene Ontology (GO) categories.

Figure S7. Number of transcripts in each cluster of orthologous groups (COG) in *E.sinensis*.

Figure S8. Top 20 transcriptional factors (TFs) predicted in Chinese mitten crab *E.sinensis* (A) and water flea *D. pulex* (B).

Figure S9. Eight clusters of differentially expressed genes (DEGs) in Chinese mitten crab hepatopancreas among four different molting stages.

Figure S10. Expression profiles of 12 differentially expressed genes from RNA-Seq (blue) and qRT-PCR (red) with *β-actin* (A) and *ubiquitin-conjugating enzyme* (B) as reference genes in different molting stages.

Table S1. Sampling information of Chinese mitten crab. PoM – promolt, InM – intermolt, PrM – promolt.

ID	Shell length (cm)	Shell width (cm)	Weight (g)	Days after molting
PoM-1	14.77	16.70	2.18	2
PoM-2	24.12	26.15	8.83	2
PoM-3	18.55	20.68	3.88	2
InM-I-1	14.89	16.12	1.84	10
InM-I-2	14.78	16.12	1.90	10
InM-I-3	22.29	24.85	6.49	10
InM-II-1	13.15	14.78	2.03	20
InM-II-2	16.16	17.75	2.74	20
InM-II-3	16.07	17.86	2.52	20
PrM-1	16.43	18.92	2.70	30
PrM-2	18.62	21.32	4.02	30
PrM-3	18.68	20.28	3.38	30

Table S2. qRT-PCR primers and amplification information for reference and target genes.

Reference/Target gene	Primer Sequence(5'-3')	Amplicon length (bp)	Efficiency (%)	correlation value (R^2)
β-actin	F: TCATCACCATCGGCAATGA<br>R: TTGTAAGTGGTCTCGTGGATG	100	100	0.999
alpha-tubulin	F:GTGGAGATCTGGCCAAGGTG<br>R: CCCACATACCAGTGCACGAA	136	100	0.999
ubiquitin-conjugating enzyme	F: TTGCGTTCACAACTCGTATCTACC<br>R:GTCCGTGAGGAGGGAACAGA	137	99.7	0.998
casein kinase isoform epsilon	F: GGGTCACGGAATCAGGAGAA<br>R: CGACGGTAGTGTCGTGTCGT	103	95.3	0.997
myosin isoform e	F: CTCGGCAGGCAAGTTTGTG<br>R: GATCTGAAGGGTTCGCTGGTC	82	97.5	0.998
long chain fatty acid ligase acsbg2	F: GTATGCATGGGTGGCAGACA<br>R: TGATGAACAGGAAGCCGTCA	136	99.5	0.999
loc 100186072	F: AAAGCTGGCCATGTCCTACG<br>R: GTGTGAGGAGGACACGCTGA	112	99.3	0.998
phosphoenolpyruvate carboxykinase	F: CCTTGATTGGCAGCATCCTC<br>R: GAGGTCCGTGATCTTGCCAT	116	96.4	0.997
methyltransferase	F: CATGGGTGGTGGCACCTAAA<br>R:TTGCAGCTCTCTCCCTCTGAA	126	98.7	0.989
juvenile hormone-inducible protein	F: GCAGCAGCGAAGAACTTCCA<br>R:CAAACAAGGAGAGATATCGAGAACG	138	98.6	0.998
loc 100880818	F: GACCTCAACGACGACATCTTC<br>R:GCCTCCTCCACATCACTCT	155	95.6	0.996
cuticle protein cbm	F:CTGTTGCCTCATCCCGAAAA<br>R:ATTGTACTCCCAGTTGCATGTCAC	129	99.6	0.998
metalloreductase steap4	F:GCCTCTCTCACCACCAGGAA<br>R:CTGTCCAGCCAGAGAGGGAA	126	96.1	0.997
ecdysone receptor	F:GCCACACTTCAACGAGAAGA<br>R:GCTGTTTCCAAACACAATGC	120	101	0.998
beta-lactamase	F: GGGTTCCGATTTAGTGCTTTA<br>R:GGTTGAGTGTTGTTCCAGTT	166	102	0.999

Table S3. Total number of transcripts and the number of transcripts with FPKM ≥1 in four different molting stages.

	Total number of transcripts	Number of transcripts with FPKM≥1
Postmolt (Day 2)	142,343	96,325
Intermolt-I (Day 10)	113,599	79,273
Intermolt-II (Day 20)	136,801	92,759
Premolt (Day 30)	151,932	104,104

Table S4. Total number of reads and the numbers of reads mapped respectively to the reference transcriptome and the corresponding individual transcriptome

	Total number of reads (million)	Number of reads that mapped to reference transcriptome (million)	Number of reads that mapped to individual reference transcriptome (million)
Postmolt (Day 2)	70.13	62.49	57.40
Intermolt-I (Day 10)	61.79	52.14	51.84
Intermolt-II (Day 20)	61.25	53.99	52.00
Premolt (Day 30)	58.54	52.22	50.66

Figure S1.

Ranking Order (Better--Good--Average)

Method	1	2	3	4
Delta CT	alpha-tublin	ubiquitin-conjugating	β-actin	GAPDH
BestKeeper	ubiquitin-conjugating	alpha-tublin	GAPDH	β-actin
Normfinder	alpha-tublin	ubiquitin-conjugating	β-actin	GAPDH
Genorm	alpha-tublin | ubiquitin-conjugating		β-actin	GAPDH
**Recommended comprehensive ranking**	**alpha-tublin**	**ubiquitin-conjugating**	**β-actin**	**GAPDH**

Gene stability by BestKeeper

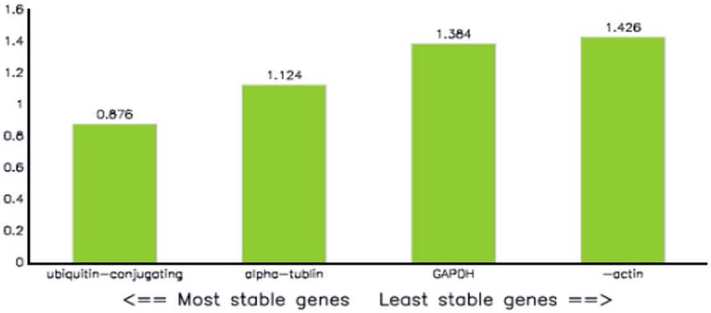

Gene name	Stability value
alpha-tublin | ubiquitin-conjugating	0.768
β-actin	1.065
GAPDH	1.331

Gene stability by Genorm

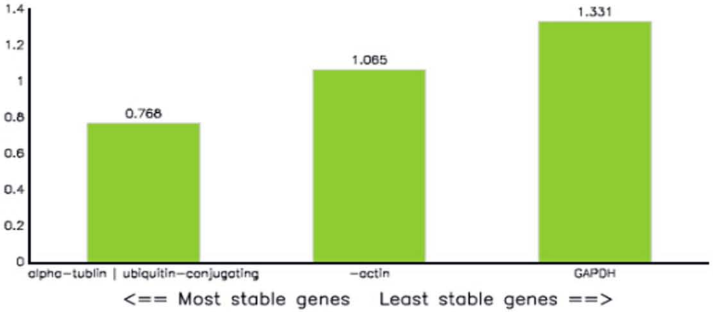

Gene name	Stability value
alpha-tublin	0.384
ubiquitin-conjugating	0.502
β-actin	1.233
GAPDH	1.426

Gene stability by normFinder

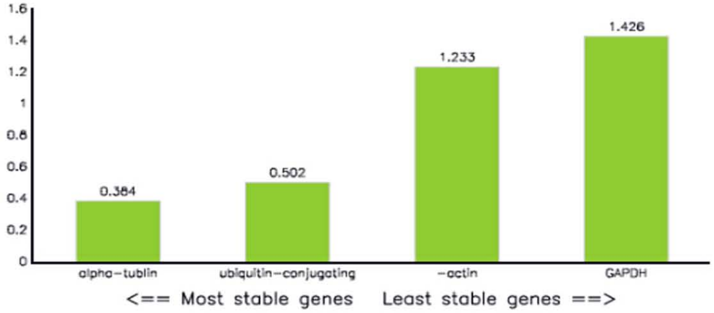

Genes	Average of STDEV
alpha-tublin	1.10
ubiquitin-conjugating	1.16
β-actin	1.47
GAPDH	1.60

Gene stability by Delta CT method

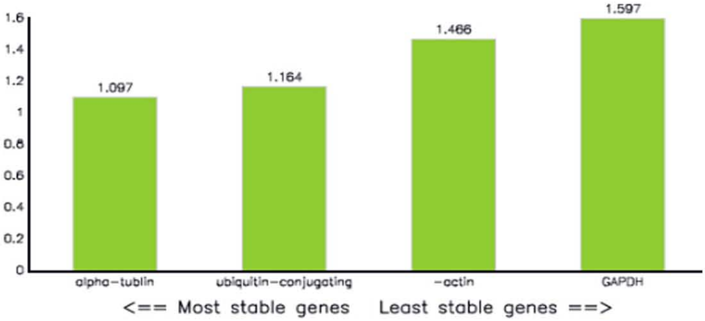

Figure S2.

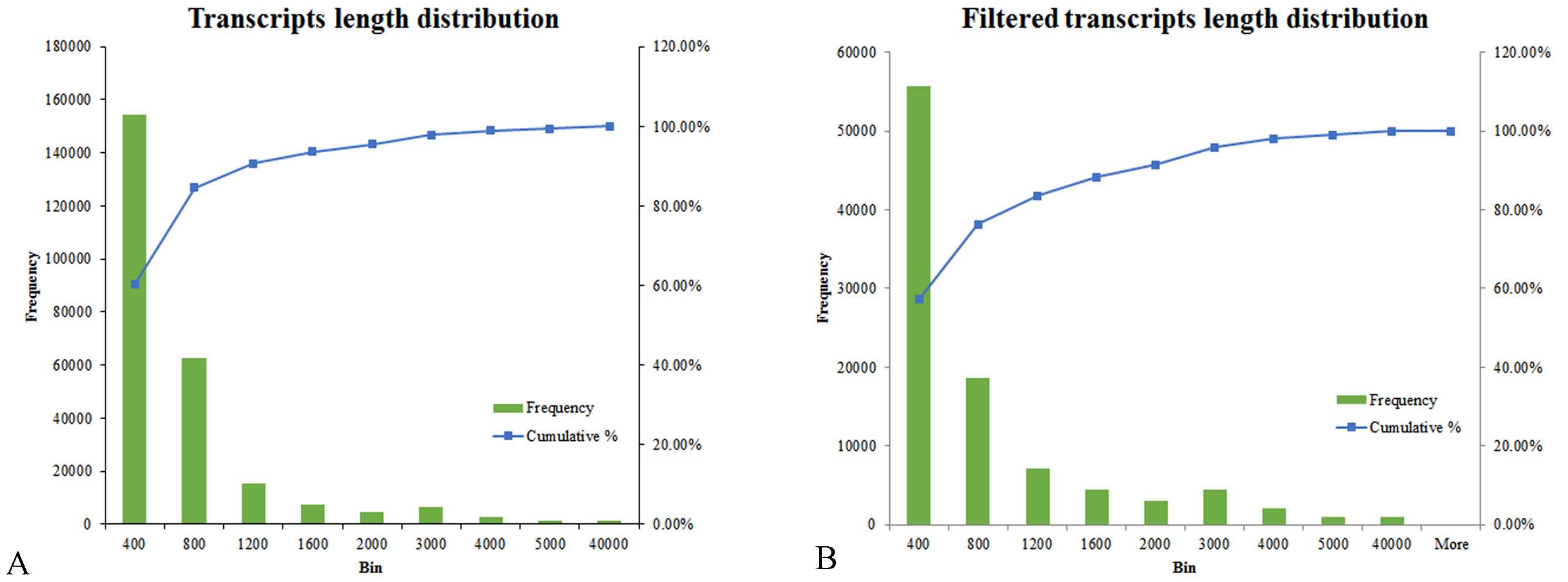
Transcripts length distribution
Frequency
Bin
Cumulative %
180000
160000
140000
120000
100000
80000
60000
40000
20000
0
120.00%
100.00%
80.00%
60.00%
40.00%
20.00%
0.00%
400
800
1200
1600
2000
3000
4000
5000
40000
A
Filtered transcripts length distribution
60000
50000
40000
30000
20000
10000
More
B

Figure S3.

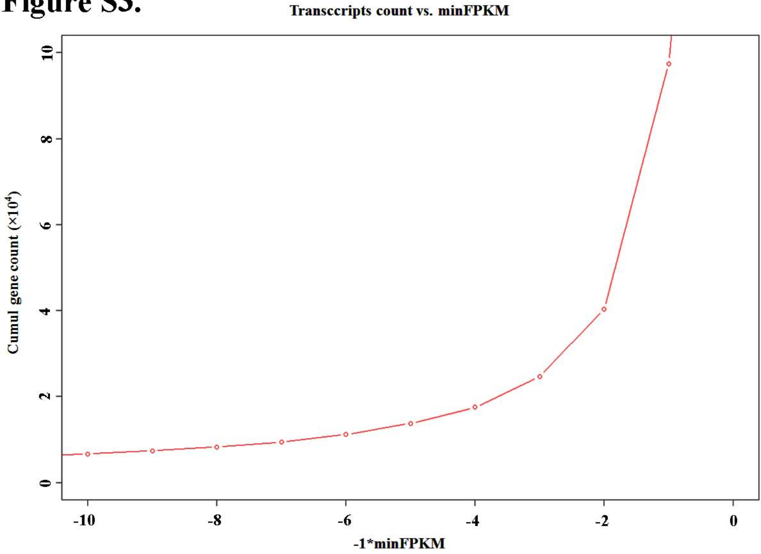
Transccripts count vs. minFPKM
Cumul gene count (×10^4)
0
2
4
6
8
10
-10
-8
-6
-4
-2
0
-1*minFPKM

Figure S4.

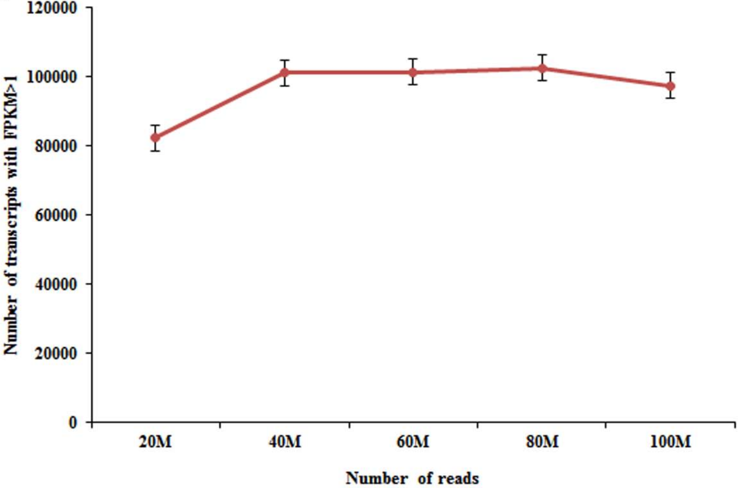

120000
100000
80000
60000
40000
20000
0
Number of transcripts with FPKM>1
20M
40M
60M
80M
100M
Number of reads

Figure S5.

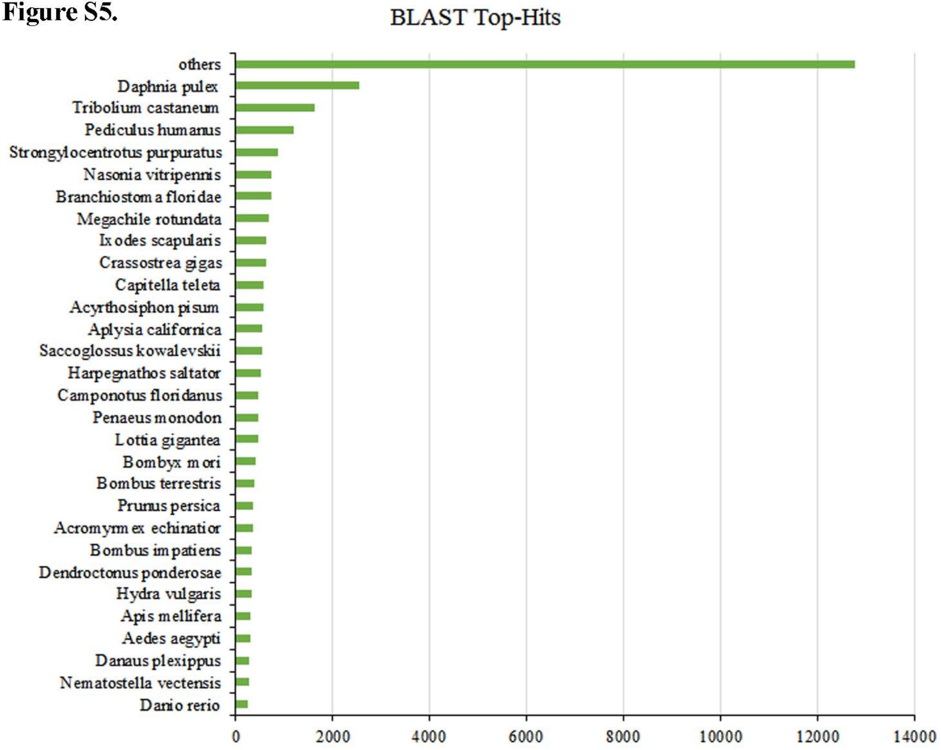
BLAST Top-Hits
others
Daphnia pulex
Tribolium castaneum
Pediculus humanus
Strongylocentrotus purpuratus
Nasonia vitripennis
Branchiostoma floridae
Megachile rotundata
Ixodes scapularis
Crassostrea gigas
Capitella teleta
Acyrthosiphon pisum
Aplysia californica
Saccoglossus kowalevskii
Harpegnathos saltator
Camponotus floridanus
Penaeus monodon
Lottia gigantea
Bombyx mori
Bombus terrestris
Prunus persica
Acromyrmex echinatior
Bombus impatiens
Dendroctonus ponderosae
Hydra vulgaris
Apis mellifera
Aedes aegypti
Danaus plexippus
Nematostella vectensis
Danio rerio
0
2000
4000
6000
8000
10000
12000
14000

Figure S6.

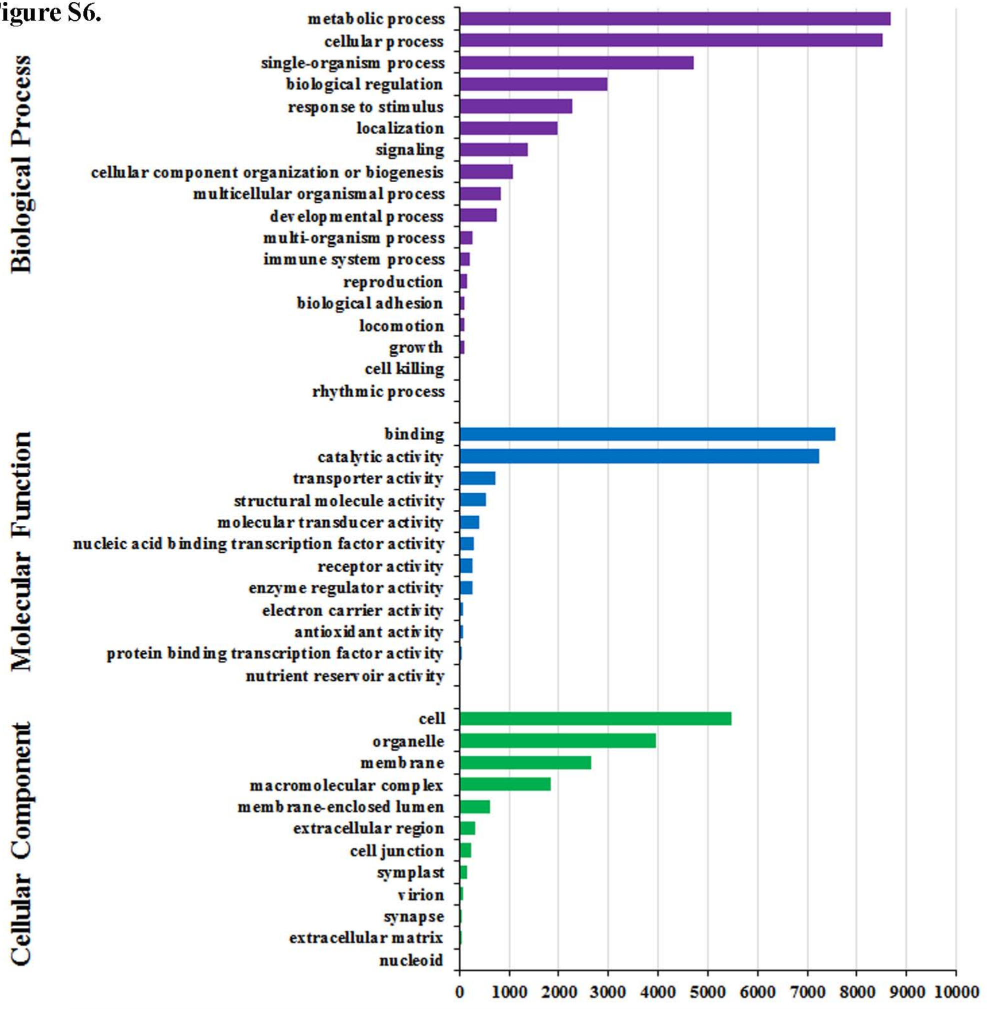
Biological Process
metabolic process
cellular process
single-organism process
biological regulation
response to stimulus
localization
signaling
cellular component organization or biogenesis
multicellular organismal process
developmental process
multi-organism process
immune system process
reproduction
biological adhesion
locomotion
growth
cell killing
rhythmic process
Molecular Function
binding
catalytic activity
transporter activity
structural molecule activity
molecular transducer activity
nucleic acid binding transcription factor activity
receptor activity
enzyme regulator activity
electron carrier activity
antioxidant activity
protein binding transcription factor activity
nutrient reservoir activity
Cellular Component
cell
organelle
membrane
macromolecular complex
membrane-enclosed lumen
extracellular region
cell junction
symplast
virion
synapse
extracellular matrix
nucleoid
0
1000
2000
3000
4000
5000
6000
7000
8000
9000
10000

Figure S7.

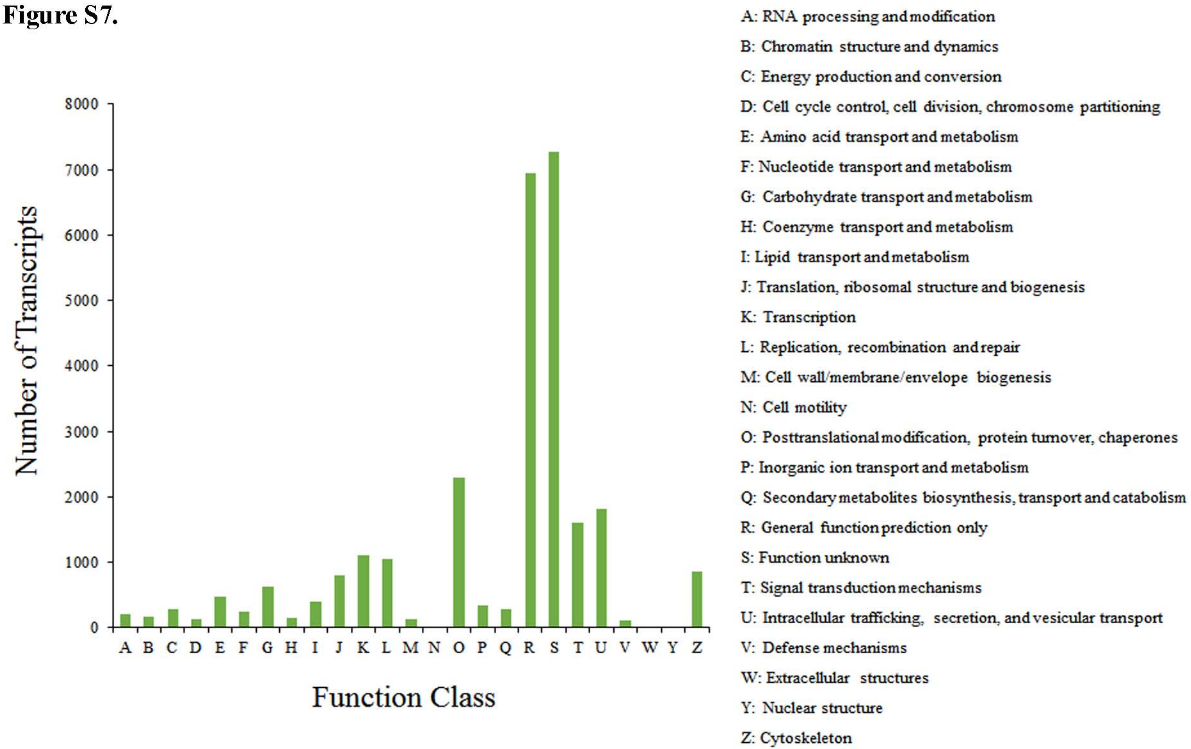

Number of Transcripts
8000
7000
6000
5000
4000
3000
2000
1000
0
A B C D E F G H I J K L M N O P Q R S T U V W Y Z
Function Class
A: RNA processing and modification
B: Chromatin structure and dynamics
C: Energy production and conversion
D: Cell cycle control, cell division, chromosome partitioning
E: Amino acid transport and metabolism
F: Nucleotide transport and metabolism
G: Carbohydrate transport and metabolism
H: Coenzyme transport and metabolism
I: Lipid transport and metabolism
J: Translation, ribosomal structure and biogenesis
K: Transcription
L: Replication, recombination and repair
M: Cell wall/membrane/envelope biogenesis
N: Cell motility
O: Posttranslational modification, protein turnover, chaperones
P: Inorganic ion transport and metabolism
Q: Secondary metabolites biosynthesis, transport and catabolism
R: General function prediction only
S: Function unknown
T: Signal transduction mechanisms
U: Intracellular trafficking, secretion, and vesicular transport
V: Defense mechanisms
W: Extracellular structures
Y: Nuclear structure
Z: Cytoskeleton

Figure S8.

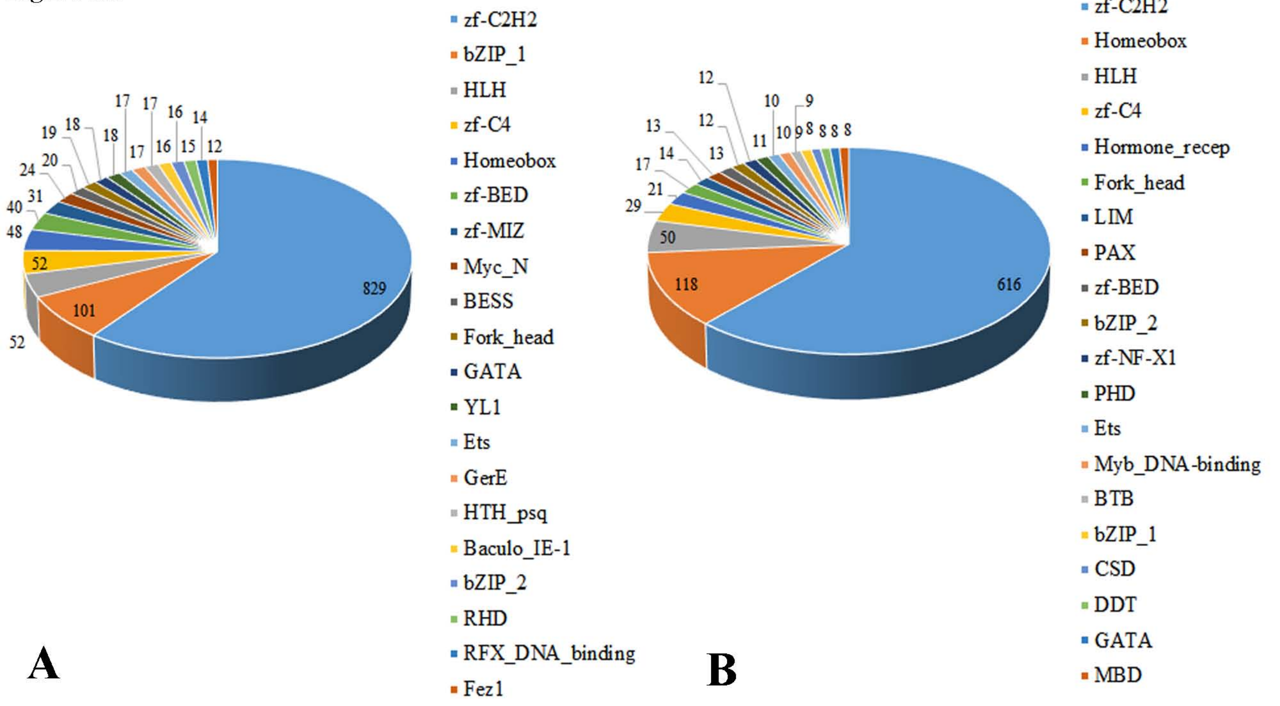
A
829
101
52
52
48
40
31
24
20
19
18
18
18
17
17
17
16
16
15
14
12
zf-C2H2
bZIP_1
HLH
zf-C4
Homeobox
zf-BED
zf-MIZ
Myc_N
BESS
Fork_head
GATA
YL1
Ets
GerE
HTH_psq
Baculo_IE-1
bZIP_2
RHD
RFX_DNA_binding
Fez1
B
616
118
50
29
21
17
14
13
13
12
12
11
10
10
9
9
8
8
8
8
zf-C2H2
Homeobox
HLH
zf-C4
Hormone_recep
Fork_head
LIM
PAX
zf-BED
bZIP_2
zf-NF-X1
PHD
Ets
Myb_DNA-binding
BTB
bZIP_1
CSD
DDT
GATA
MBD

Figure S9.

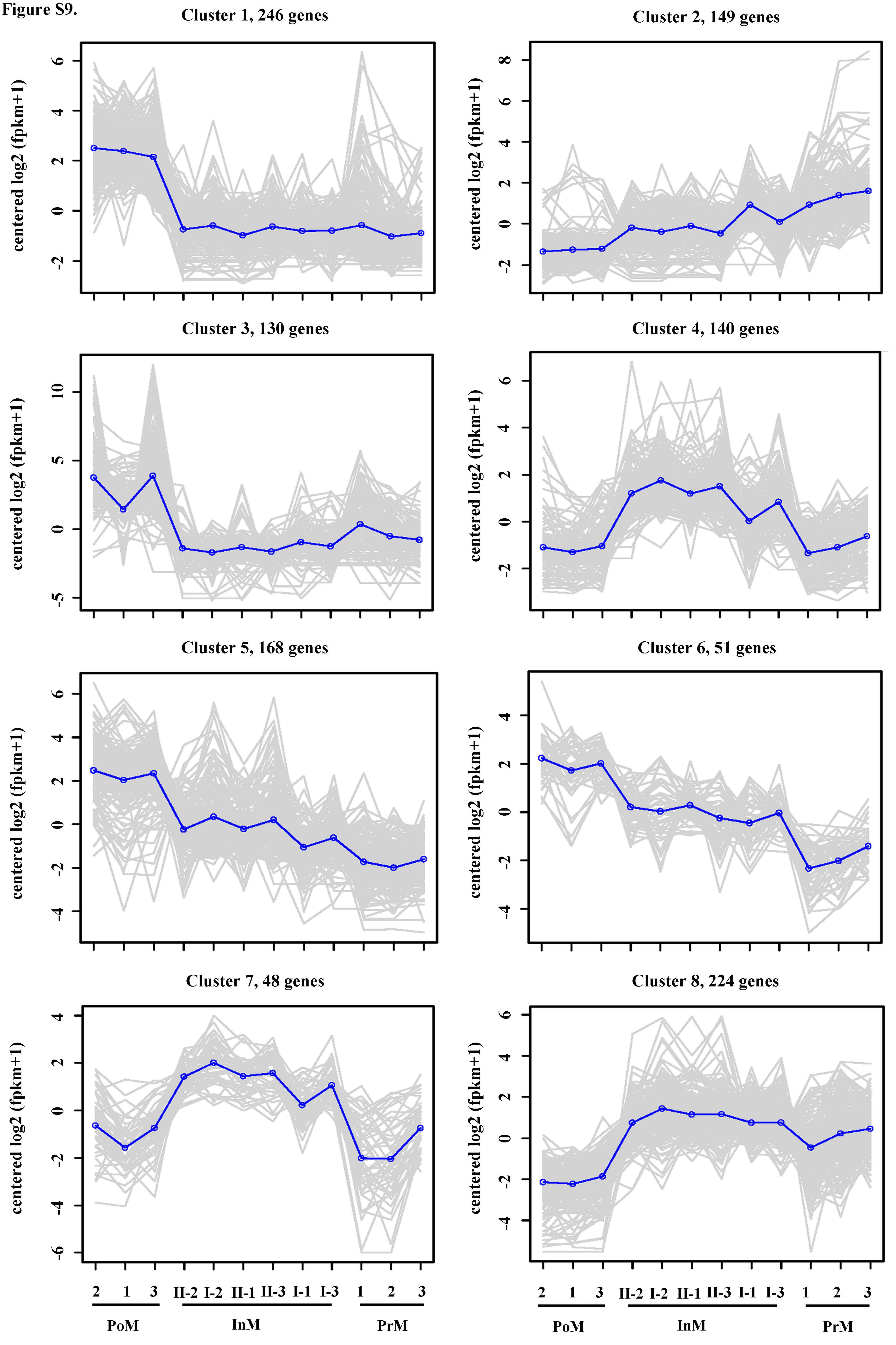

Cluster 1, 246 genes
Cluster 2, 149 genes
Cluster 3, 130 genes
Cluster 4, 140 genes
Cluster 5, 168 genes
Cluster 6, 51 genes
Cluster 7, 48 genes
Cluster 8, 224 genes
centered log2 (fpkm+1)
2 1 3 II-2 I-2 II-1 II-3 I-1 I-3 1 2 3
PoM
InM
PrM

Figure S10.

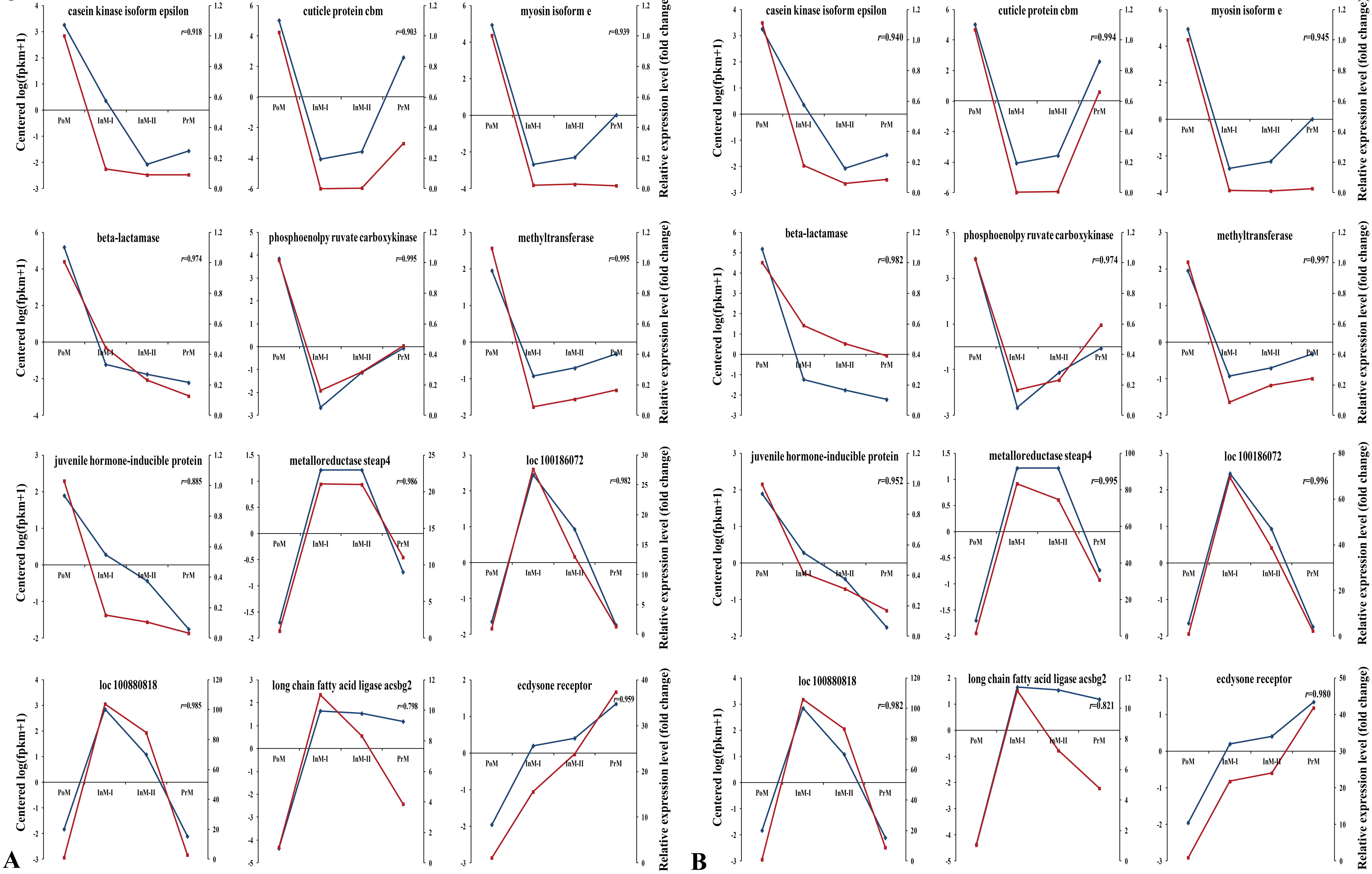
A
casein kinase isoform epsilon
r=0.918
cuticle protein cbm
r=0.903
myosin isoform e
r=0.939
beta-lactamase
r=0.974
phosphoenolpy ruvate carboxykinase
r=0.995
methyltransferase
r=0.995
juvenile hormone-inducible protein
r=0.885
metalloreductase steap4
r=0.986
loc 100186072
r=0.982
loc 100880818
r=0.985
long chain fatty acid ligase acsbg2
r=0.798
ecdysone receptor
r=0.959
B
casein kinase isoform epsilon
r=0.940
cuticle protein cbm
r=0.994
myosin isoform e
r=0.945
beta-lactamase
r=0.982
phosphoenolpy ruvate carboxykinase
r=0.974
methyltransferase
r=0.997
juvenile hormone-inducible protein
r=0.952
metalloreductase steap4
r=0.995
loc 100186072
r=0.996
loc 100880818
r=0.982
long chain fatty acid ligase acsbg2
r=0.821
ecdysone receptor
r=0.980
PoM
InM-I
InM-II
PrM
Centered log(fpkm+1)
Relative expression level (fold change)
